# A comparative study of elective nodal irradiation and involved field irradiation in elderly patients with advanced esophageal cancer

**DOI:** 10.3389/fonc.2023.1323908

**Published:** 2023-12-20

**Authors:** Yuanfei Dai, Di Huang, Wei Zhao, Jie Wei

**Affiliations:** Department of Oncology, The Affiliated Chuzhou Hospital of Anhui Medical University (The First People’s Hospital of Chuzhou), Chuzhou, Anhui, China

**Keywords:** elderly, radiotherapy, involved field irradiation, elective nodal irradiation, prognostic factor

## Abstract

**Objective:**

This comparative study aimed to explore the feasibility of involved field irradiation (IFI) in the radiotherapy of elderly patients with advanced esophageal cancer, compared with elective nodal irradiation (ENI).

**Methods:**

A total of 245 elderly patients (age ≥70 years) with advanced esophageal cancer, who received radiotherapy in our department from January 2014 to December 2020, were divided into the ENI group (n=111) and the IFI group (n=134). Clinical efficacy, toxicities, survival rates, treatment failures, and multifactorial survival analyses were conducted for both groups.

**Results:**

The ENI group and the IFI group showed no significant differences in terms of short-term efficacy (91.9% *vs* 91.0%, P=0.814), 1-year overall survival (OS) (81.1% *vs* 74.6%, P=0.228), 2-year OS (22.5% *vs* 25.4%, P= 0.603), 1-year progression-free survival (PFS) (56.8% *vs* 51.5%, P= 0.198), 2-year PFS (8.1% *vs* 9.0%, P=0.814), regional failures (38.7% *vs* 31.3%, P=0.226), and distant metastasis (21.6% *vs* 14.9%, P=0.174). The median overall survival (OS) was 19 months in the ENI group and 18 months in the IFI group (Log-rank*χ* ^2^ = 0.012, P=0.913). The median progression-free survival (PFS) was 13 months in the ENI group and 11 months in the IFI group (Log-rank*χ* ^2^ = 1.834, P=0.176). There were no significant statistical differences in both OS and PFS (P>0.05). The incidence of grade ≥3 radiation pneumonia and grade ≥3 radiation esophagitis in the IFI group was 8.2% and 11.2%, respectively, which were significantly lower than those in the ENI group (17.1%, P=0.034; 21.6%, P=0.026). Univariate analysis revealed that age, gender, T stage, N stage, and synchronous chemotherapy were factors affecting prognosis. Multivariate analysis showed that age, gender, T stage, and synchronous chemotherapy were independent prognostic factors, with hazard ratios of 1.227, 1.466, 2.441, and 2.714, and P values of <0.001, 0.006, <0.001, and<0.001, respectively.

**Conclusion:**

IFI is a suitable choice for elderly patients with advanced esophageal cancer, as it yields similar efficacy to ENI while reducing toxicities. Age, gender, T stage, and synchronous chemotherapy are independent prognostic factors for elderly patients with esophageal cancer.

## Introduction

Esophageal cancer ranks as the 8th most commonly diagnosed cancer and the 6th leading cause of cancer-related deaths (1). The treatment of esophageal cancer includes surgery, radiotherapy, and chemotherapy. However, due to the stealthy onset of the disease, most patients are diagnosed at an advanced stage, missing the opportunity for surgical treatment. Therefore, definitive concurrent chemoradiotherapy has become the recommended treatment for locally advanced esophageal cancer (2). With the aging population advancing, it is expected that by 2030, elderly patients will account for 70% of cancer patients in the United States (3). Especially for patients aged 70 and above, due to declining physical condition and multiple comorbidities, they are often excluded from many clinical studies. Precise radiotherapy delineation models for esophageal cancer mainly include elective nodal irradiation (ENI) and involved field irradiation (IFI), but there is currently no clear consensus on which way to choose, especially for elderly patients. This study conducted a retrospective analysis of elderly patients with advanced-stage esophageal cancer who underwent ENI and IFI in our hospital. The study aimed to compare the clinical efficacy, toxicities, and survival rates of patients treated with different irradiation techniques. Additionally, the factors contributing to the failure of radiotherapy in elderly patients with advanced-stage esophageal cancer were analyzed. The feasibility of using involved-field irradiation in the radiotherapy of elderly patients with advanced-stage esophageal cancer was explored. Furthermore, the study investigated the impact of various factors on the survival prognosis of elderly patients with advanced-stage esophageal cancer.

## Materials and methods

### Patients

A retrospective study was conducted on 245 patients with esophageal cancer who underwent radiotherapy in our department from January 2014 to December 2020. The inclusion criteria were as follows: newly diagnosed patients, pathological diagnosis of esophageal squamous cell carcinoma, stage II-III disease(The eighth edition of AJCC), Karnofsky Performance Score (KPS) ≥70, age ≥70 years, complete hospitalization data and relevant examinations, no esophageal perforation or signs of perforation before treatment, no severe internal medicine diseases or other malignancies, all patients received radical intensity-modulated radiotherapy (IMRT), and signed informed consent for radiochemotherapy. This study was approved by the Institutional Review Board of Anhui Medical University (Approval No. 81220097) prior to patient enrollment. According to the delineation range of the clinical target area, the patients were divided into two groups: the elective nodal irradiation (ENI) group with 111 cases and the involved field irradiation (IFI) group with 134 cases. There was no statistically significant difference in baseline characteristics between the two groups (P>0.05), as shown in **Table 1**.

**Table 1:** Comparison of basic data between the two groups.

Content	ENI (111)	IFI (134)	*χ* ^2^	P
Gender				
male	52	64	0.020	0.887
female	59	70		
Age				
<80 years	87	104	0.021	0.885
≥80 years	24	30		
Length				
<5cm	59	71	0.001	0.979
≥5cm	52	63		
T stage				
T1-2	65	80	0.033	0.856
T3-4	46	54		
N stage				
N0	51	59	0.090	0.764
N+	60	75		
Chemotherapy				
+	29	39	0.269	0.604
–	82	95		

### Chemotherapy

(1) In general, patients with a good general condition undergo concurrent chemoradiotherapy, typically using the TP regimen, which consists of paclitaxel 175mg/m^2^ D1 and cisplatin 20mg/m^2^ D1-D3. For individuals who cannot tolerate this regimen, monotherapy with oral S-1 chemotherapy is administered.(2) For elderly patients with multiple comorbidities and poor physical condition, they undergo radiotherapy alone.

### Radiotherapy

Radiotherapy was delivered using IMRT and divided into the IFI group and ENI group. In the ENI group: (1) Tumor volume (GTV). GTV-T represented the visible tumor. GTV-N included positive metastatic lymph nodes, with a longitudinal diameter ≥0.5 cm in the CT axial images, located adjacent to the esophagus, in the tracheoesophageal groove, at the angle of the diaphragm, and in the abdominal lymph nodes, or with a longitudinal diameter ≥1 cm in other regions, or with multiple (≥5) small lymph nodes clustered together; PET-CT showed high metabolic activity, with a standardized uptake value (SUV) ≥2.5. (2) CTV-T extended 3 cm above and below the GTV-T, and 0.5 cm anteriorly, posteriorly, and laterally. CTV-N included: (a) Upper thoracic segment: lymph nodes in the lower neck, groups 1, 2, 4, 5, and 7, and lymph nodes in the area of the positive lymph nodes. (b) Middle thoracic segment: lymph nodes in groups 2, 4, 5, and 7, and lymph nodes in the area of the positive lymph nodes. (c) Lower thoracic segment: lymph nodes in groups 4, 5, 7, para-aortic region, left gastric artery region, and lymph nodes in the area of the positive lymph nodes. (3) PTV extended 1 cm above and below the CTV, and 0.5 cm anteriorly, posteriorly, and laterally. In the IFI group: The delineation of GTV-T, GTV-N, CTV-T, and PTV were the same as in the ENI group. However, CTV-N only included the regions of the positive lymph nodes, without prophylactic irradiation of the corresponding lymph drainage regions. The PTV in both groups extended 1 cm above and below the CTV, and 0.5 cm anteriorly, posteriorly, and laterally. The radiation was delivered using 6MV X-rays, with a dose of 60-66 Gy/30-33 fractions for PTV in the IFI group, and a dose of 60-66 Gy/30-33 fractions for PTV1 (primary lesions and metastatic lymph nodes of esophageal cancer) and a dose of 50-54 Gy/30-33 fractions for PTV2 (lymph drainage regions) in the ENI group.

### Follow-up

After completion of treatment, all patients are followed up through telephone calls and clinic visits. The first follow-up visit is scheduled one month after the completion of treatment, followed by follow-up visits every three months during the first year, every six months during the second year, and annually thereafter.

### Efficacy and toxicity

Patients underwent follow-up evaluations, including CT scans and barium meals, 1 month after the completion of radiotherapy to assess treatment efficacy. The RECIST (4) criteria were utilized for evaluation, specifically: complete response (CR), partial response (PR), stable disease (SD), and progressive disease (PD). The response rate was calculated as CR + PR, and the disease control rate was calculated as CR + PR + SD. Toxicity were assessed using the RTOG criteria.

### Treatment failure

Categorization of treatment failure includes regional failure (including in-field failure and out-of-field failure) and distant metastasis. Failure to control or recurrence of the primary esophageal lesion and regional lymph nodes is referred to as regional failure, with failures within the radiation field classified as in-field failure and failures outside the radiation field classified as out-of-field failure. The presence of metastasis beyond the primary esophageal lesion and regional lymph nodes is termed distant metastasis.

### Statistical analyses

Data analysis was performed using SPSS 26.0. Chi-square test was applied for analyzing count data. Kaplan-Meier method was used for calculating and plotting survival curves. Log-rank test was conducted for univariate analysis of survival prognosis. Cox model was employed for multivariate analysis of prognosis. A p-value<0.05 was considered statistically significant for detecting differences.

## Results

### Recent efficacy

The recent efficacy evaluation was conducted 3 months after the completion of radiotherapy. The efficacy assessment showed that the ENI group had an effectiveness rate of 91.9% (102/111), while the IFI group had an effectiveness rate of 91.0% (122/134). There was no statistically significant difference between the two groups (P=0.814), as shown in **Table 2**.

**Table 2:** Comparison of recent efficacy between the two groups.

	ENI	IFI	*χ* ^2^	P
CR+PR	102 (91.9%)	122 (91.0%)	0.056	0.814
SD+PD	9 (8.1%)	12 (9.0%)		

### Comparisons of OS and PFS

In the 245 patients in the two groups, the median overall survival (OS) was 18 months (95%CI:16.987-19.013), and the median progression-free survival (PFS) was 12 months (95%CI:11.107-12.893). The 1-year and 2-year survival rates were 77.6% and 24.1%, respectively. The 1-year and 2-year progression-free survival rates were 48.6% and 8.6%, respectively. There were no statistically significant differences in the 1-year and 2-year survival rates or progression-free survival rates between the two groups (P>0.05), as shown in **Table 3**.

**Table 3:** Comparison of survival rate and progression free survival rate between the two groups.

	1 year OS	2 years OS	1 year PFS	2 years PFS
ENI	81.1%	22.5%	56.8%	8.1%
IFI	74.6%	25.4%	51.5%	9.0%
*χ* ^2^	1.453	0.270	1.656	0.056
P	0.228	0.603	0.198	0.814

**Figure 1:**
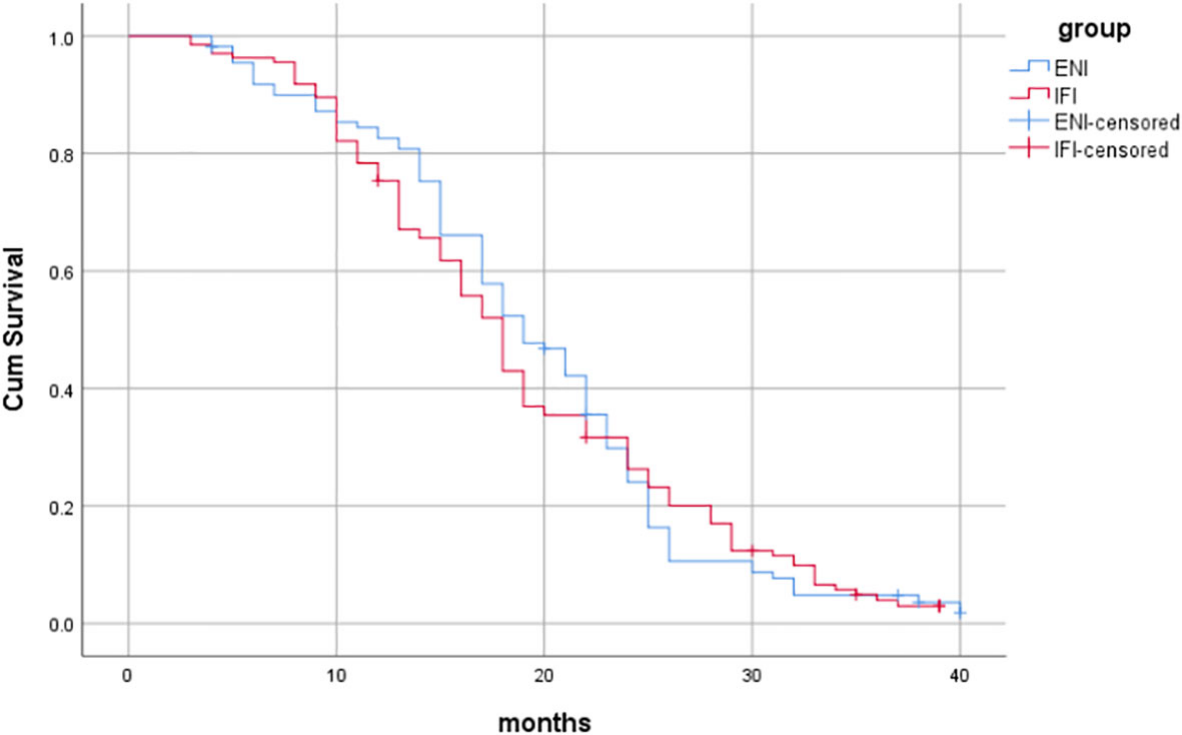
Comparison of OS between the two groups.

**Figure 2:**
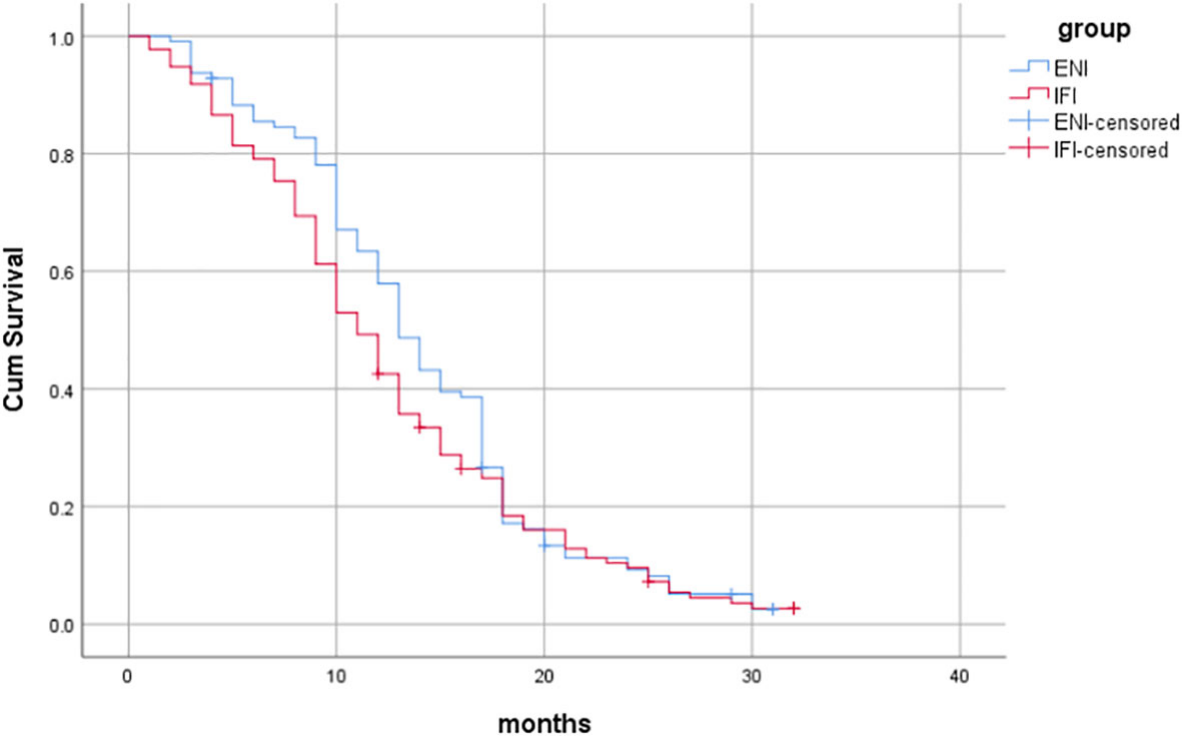
Comparison of PFS between the two groups.

Among the patients, 5 cases died due to other internal diseases, including 3 cases in the ENI group (1 case with coronary heart disease and heart failure, and 2 cases with lung infection) and 2 cases in the IFI group (1 case with chronic obstructive pulmonary disease and 1 case with lung infection). The median OS was 19 months (95%CI: 16.610-21.390) in the ENI group and 18 months (95%CI: 16.687-19.313) in the IFI group, with no statistically significant difference (Log-rank*χ* ^2^ = 0.012, *P*=0.913), as shown in **Figure 1**. The median PFS was 13 months (95%CI: 11.723-14.277) in the ENI group and 11 months (95%CI: 9.639-12.361) in the IFI group, with no statistically significant difference (Log-rank*χ* ^2^ = 1.834, *P*=0.176), as shown in **Figure 2**.

### Analysis of adverse reactions

During the treatment process, the incidence of grade ≥3 acute hematological toxicity in the ENI group and the IFI group was 24.3% (27/111) and 18.7% (25/134), respectively, with no statistically significant difference (P=0.280). The incidence of grade ≥3 radiation pneumonitis in the ENI group and the IFI group was 17.1% (19/111) and 8.2% (11/134), respectively, showing a statistically significant difference (P=0.034). The incidence of grade ≥3 radiation esophagitis in the ENI group and the IFI group was 21.6% (24/111) and 11.2% (15/134), respectively, showing a significant difference (P=0.026), as shown in **Table 4**.

**Table 4:** Comparison of radiation toxicity between the two groups.

Irradiation toxicities	ENI group (41)	IFI group (47)	*χ* ^2^	P
Myelosuppression				
Grade 0	41	70	1.166	0.280
Grade 1-2	43	39		
Grade 3-4	27	25		
Pneumonitis				
Grade 0	66	101	4.483	0.034
Grade 1-2	26	22		
Grade 3-4	19	11		
Andesophagitis				
Grade 0	33	68	4.932	0.026
Grade 1-2	54	51		
Grade 3-4	24	15		

### Analysis of treatment failure

In the ENI group, there were 51 cases of treatment failure, with 43 cases of regional failure, 24 cases of distant metastasis, and 16 cases of both regional failure and distant metastasis. In the IFI group, there were 50 cases of treatment failure, with 42 cases of regional failure, 20 cases of distant metastasis, and 12 cases of both regional failure and distant metastasis. There was no statistically significant difference in treatment failure between the two groups (P>0.05), as shown in **Table 5**.

**Table 5:** Comparison of treatment failure between the two groups.

Failure mode	ENI group (111)	IFI group (134)	*χ* ^2^	P
Regional failure	43	42	1.465	0.226
Primary in-field	22	26	0.007	0.935
Lymph node in-field	15	11	1.801	0.180
out-of-field failure	6	5	0.397	0.529
Distant metastasis	24	20	1.848	0.174
Regional failure and distant metastasis	16	12	1.787	0.181
Total failure	51	50	1.867	0.172

### Analysis of prognostic factors for survival

**Figure 3:**
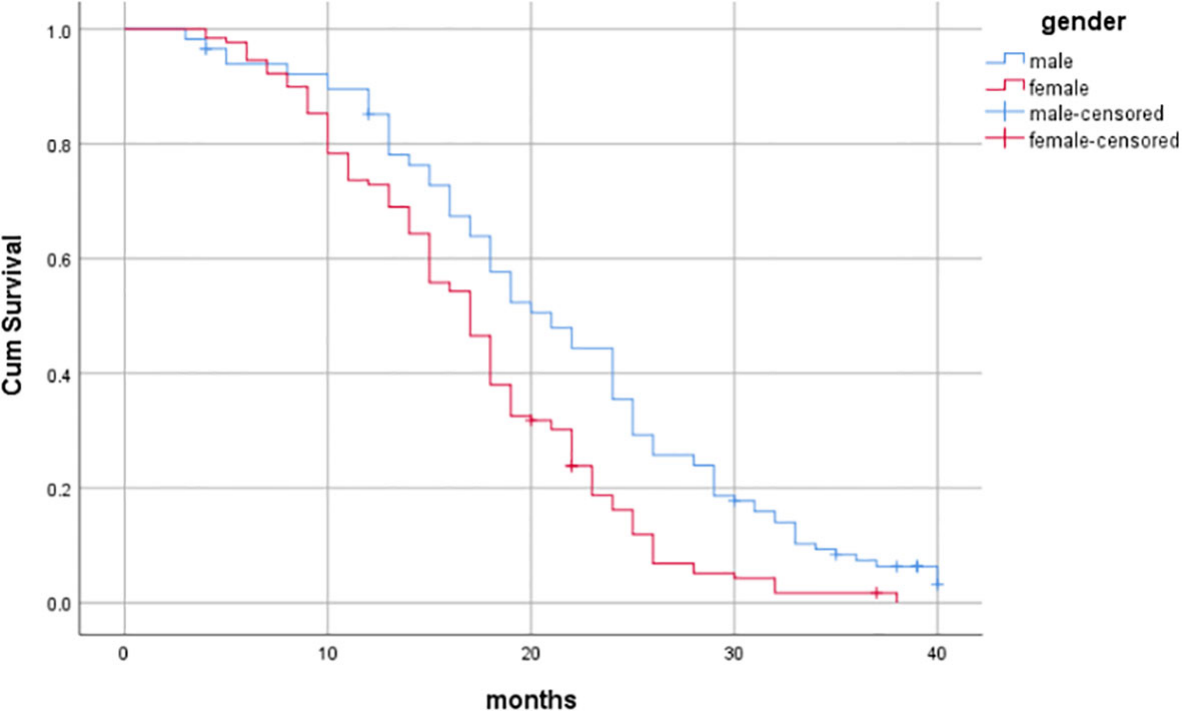
Influence of gender on prognosis of patients.

**Figure 4:**
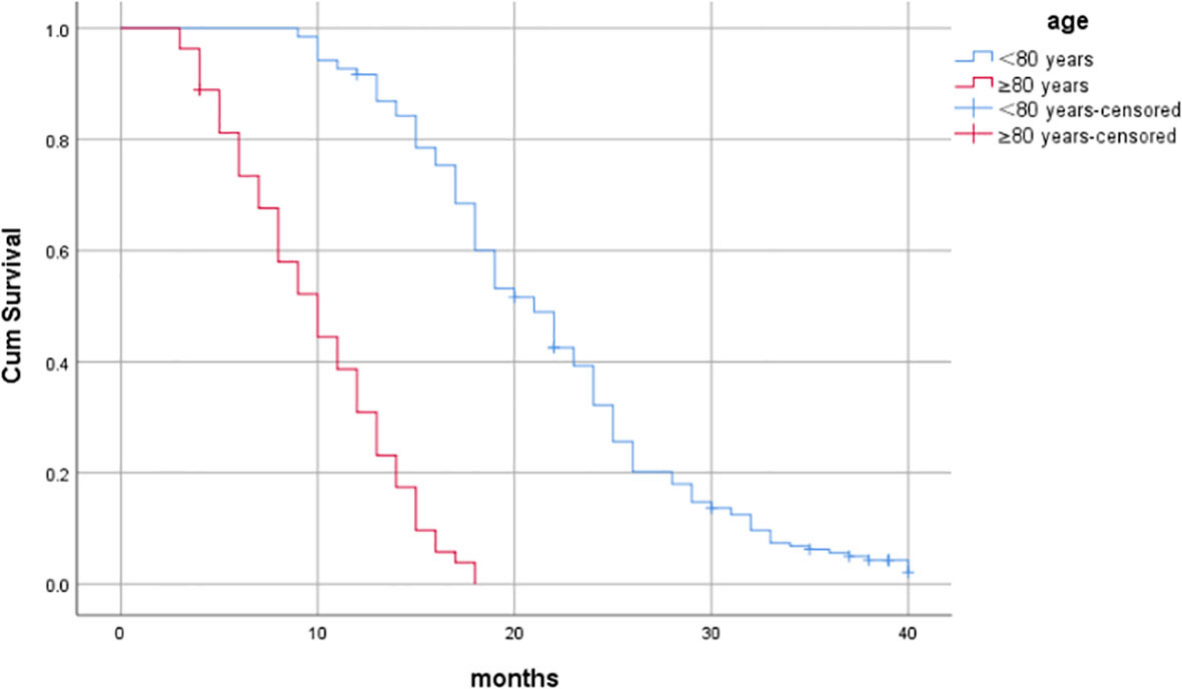
Influence of age on prognosis of patients.

**Figure 5:**
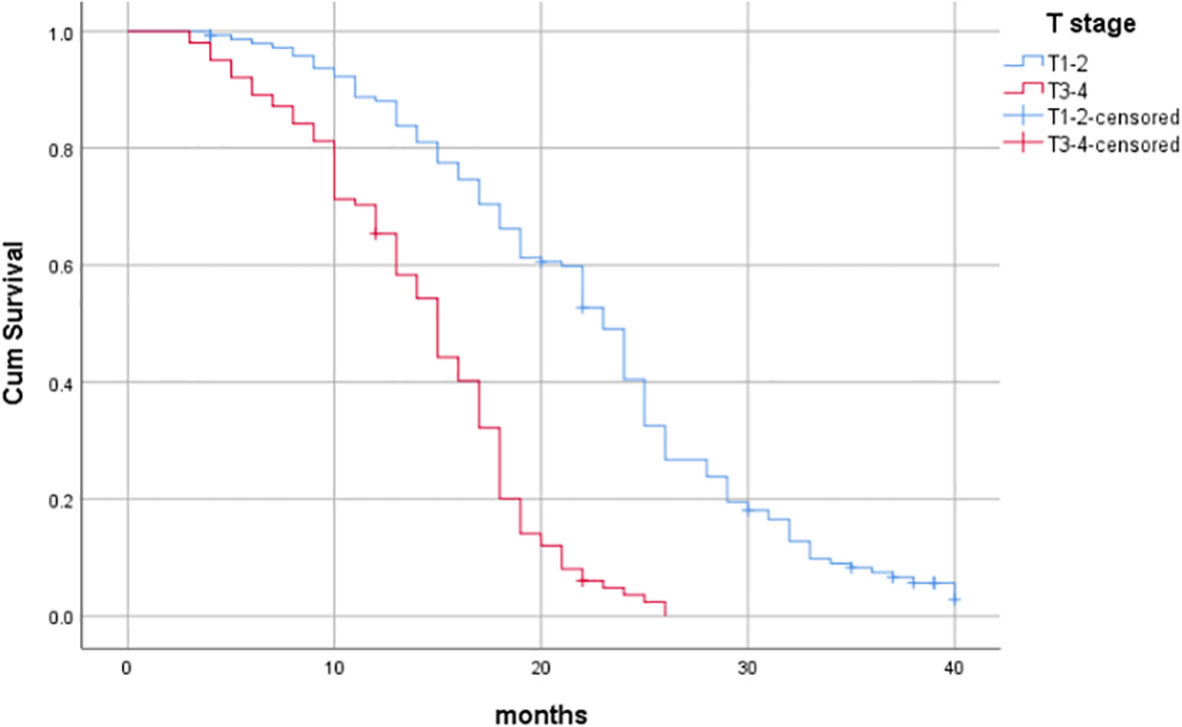
Influence of T stage on prognosis of patients.

**Figure 6:**
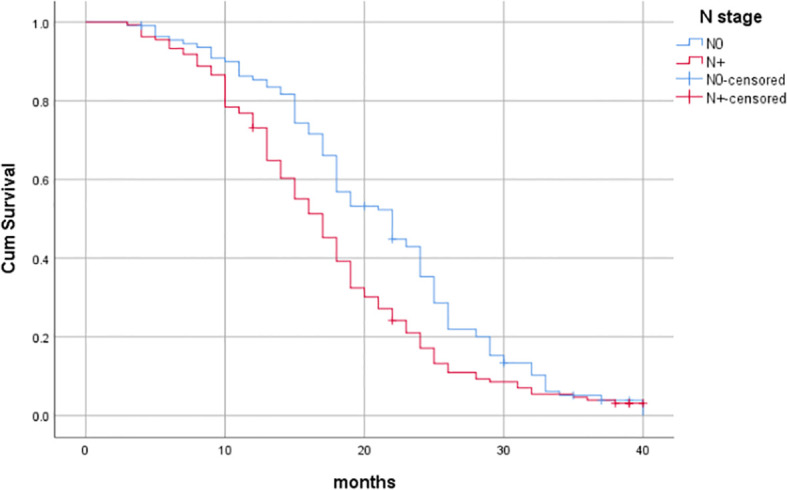
Influence of N stage on prognosis of patients.

**Figure 7:**
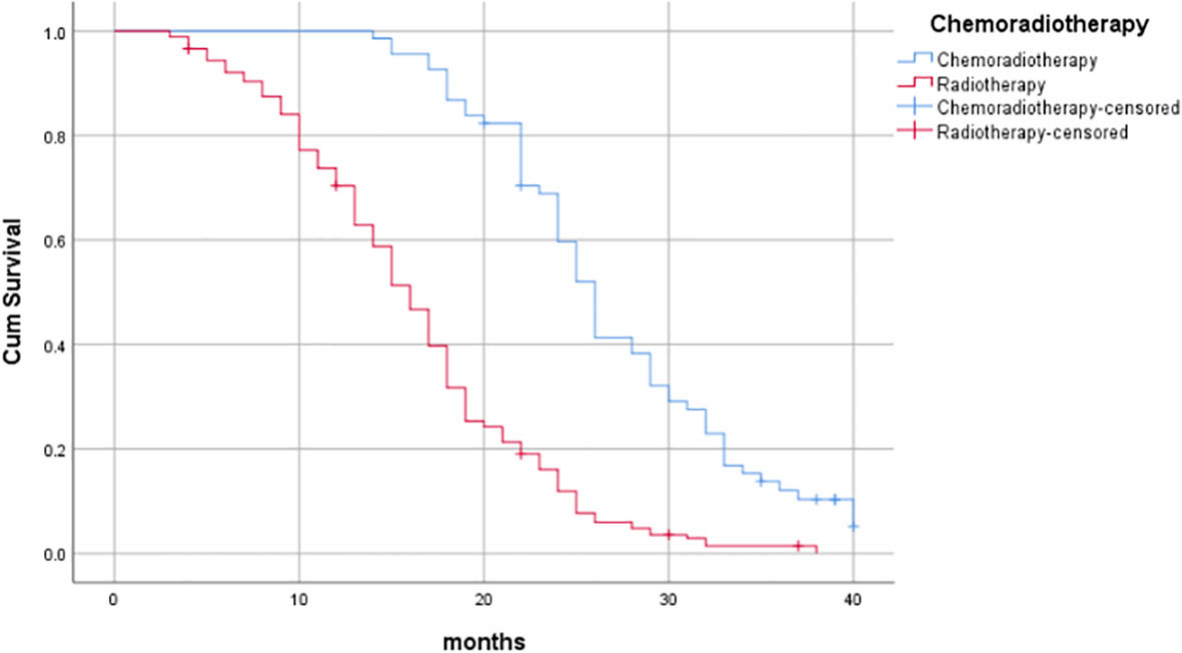
Influence of chemoradiotherapy on prognosis of patients.

Through univariate analysis of patient survival, it was found that gender, age, T stage, N stage, and whether synchronous chemotherapy had statistically significant effects on overall survival (P<0.05). Specifically, the median overall survival (OS) for males and females (gender) was 21 months (95%CI:18.231-23.769) and 17 months (95%CI: 15.552-18.448), respectively (Log-rank*χ* ^2^ = 17.993, *P*<0.001); for patients <80 years and ≥80 years (age), the median OS was 21 months (95%CI: 19.372-22.628) and 10 months (95%CI: 8.002-11.998), respectively (Log -rank*χ* ^2^ = 183.937, *P*<0.001); for T1-2 and T3-4 (T stage), the median OS was 23 months (95%CI: 21.725-24.275) and 15 months (95%CI: 13.617 -16.383), respectively (Log-rank*χ* ^2^ = 80.056, *P*<0.001); for N0 and N+ (N stage), the median OS was 22 months (95%CI: 18.899-25.101) and 17 months (95%CI: 15.397-18.603), respectively (Log-rank*χ* ^2^ = 9.411, *P*=0.002); and for whether synchronous chemotherapy was performed, the median OS was 26 months (95%CI: 24.705-27.295) and 16 months (95%CI: 14.831-17.169), respectively (Log-rank*χ* ^2^ = 68.676, *P*<0.001), as shown in **Table 6** and **Figures 3**–**7**.

**Table 6:** Univariate analysis of prognosis for all patients.

Factor	Cases	Median survival (months)	*χ* ^2^	P
Gender				
male	116	21 (95%CI:18.231-23.769)	17.993	<0.001
female	129	17 (95%CI:15.552-18.448)		
Age				
<80 years	191	21 (95%CI:19.372-22.628)	183.937	<0.001
≥80 years	54	10 (95%CI:8.002-11.998)		
T stage				
T1-2	144	23 (95%CI:21.725-24.275)	80.056	<0.001
T3-4	101	15 (95%CI:13.617-16.383)		
N stage				
N0	111	22 (95%CI:18.899-25.101)	9.411	0.002
N+	134	17 (95%CI:15.397-18.603)		
Chemoradiotherapy				
+	68	26 (95%CI:24.705-27.295)	68.676	<0.001
–	177	16 (95%CI:14.831-17.169)		

Furthermore, through multivariate Cox regression analysis, age, gender, T stage, and whether synchronous chemotherapy were identified as independent prognostic factors, with HR values of 1.227, 1.466, 2.441, and 2.714, respectively. The corresponding P-values were <0.001, 0.006, <0.001, and<0.001, as shown in **Table 7**.

**Table 7:** Multivariate analysis of prognosis for all patients.

	B	SE	Wald	df	Sig.	Exp(B)	95.0%CI for Exp(B)	
							Lower	Upper
Age	0.205	0.021	91.905	1	<0.001	1.227	1.177	1.280
Gender	0.382	0.139	7.566	1	0.006	1.466	1.116	1.924
T stage	0.893	0.166	28.827	1	<0.001	2.441	1.762	3.381
N stage	0.025	0.143	0.030	1	0.862	1.025	0.774	1.358
Chemoradiotherapy	0.998	0.164	36.932	1	<0.001	2.714	1.967	3.745

Age was analyzed based on actual years.

## Discussion

The standard treatment for locally advanced esophageal cancer patients who are unable or refuse surgery is to undergo chemotherapy, and the selection between elective nodal irradiation (ENI) and involved-field irradiation (IFI) has been controversial (5, 6). Elderly patients with esophageal cancer often have poor physical conditions, making it crucial to choose the appropriate radiotherapy method in this study. Li et al. (7) reported that among esophageal cancer patients receiving IFI irradiation, the intrafield failure rate was 69.6%, distant metastasis rate was 33.9%, and regional lymph node failure rate was only 12.5% during a median follow-up of 34 months. Ma et al. (8) conducted a comparative study on the two radiotherapy methods and found that the regional failure rates for the ENI and IFI groups were 17.6% and 13.7%, respectively, with no statistical difference (p=0.837). The lymph node out-of-field recurrence rate in the IFI group was only 2%. Furthermore, the 3-year survival rates for the ENI and IFI groups were 41.3% and 32.0%, respectively (p=0.58), and the 3-year local control rates were 85.7% and 80.1% (p=0.34), with no statistically significant differences. Wang et al. (9), in a meta-analysis of 23 studies involving 4120 patients, found no significant differences in terms of 1-year, 2-year, and 3-year local control rates and overall survival rates between the IFI and ENI groups. Zhu et al. (5) also concluded that there were no significant differences in terms of 1-year, 2-year, and 3-year survival rates, as well as rates of local recurrence, regional recurrence, and distant metastasis, between patients receiving ENI and IFI treatment. In our study, a comparison between the ENI and IFI groups showed no significant differences in terms of short-term clinical efficacy (91.9% *vs*. 91.0%, P=0.814), 1-year survival rate (81.1% *vs*. 74.6%, P=0.228), 2-year survival rate (22.5% *vs*. 25.4%, P=0.603), 1-year progression-free survival rate (56.8% *vs*. 51.5%, P=0.198), 2-year progression-free survival rate (8.1% *vs*. 9.0%, P=0.814), regional failure rate (38.7% *vs*. 31.3%, P=0.226), distant metastasis rate (21.6% *vs*. 14.9%, P=0.174), indicating no significant statistical differences (P>0.05) in terms of treatment efficacy, similar to the aforementioned studies. The overall survival rate in our study was lower than the results of the aforementioned studies, possibly due to the older age (≥70 years) and higher comorbidity rate among the study population, as well as the relatively few patients who completed concurrent chemoradiotherapy, which may have resulted in decreased treatment efficacy.

Simultaneous radiotherapy and chemotherapy for esophageal cancer patients can result in significant toxic side effects. Jing et al. (10) conducted a retrospective analysis of 137 esophageal cancer patients (including 54 in the ENI group and 83 in the IFI group), and found that the incidence of grade ≥3 acute radiation esophagitis was significantly higher in the ENI group compared to the IFI group (18.5% *vs*. 6.0%, P=0.027). Li et al. (11) analyzed 110 esophageal cancer patients (including 56 in the ENI group and 54 in the IFI group), and found that the IFI group had significantly lower incidence of grade ≥2 radiation pneumonitis and radiation esophagitis compared to the ENI group (12.9% *vs*. 26.8%, P=0.011; 20.4% *vs*. 37.5%, P=0.001). Liu et al. (12) found that the incidence of acute radiation pneumonitis (p=0.005) and hematologic toxicity (p=0.029) was significantly higher in the ENI group compared to the IFI group. Cheng et al. (13) also observed that the incidence of grade ≥3 acute esophagitis and pneumonitis was significantly lower in the IFI group compared to the ENI group. In this study, the ENI group and IFI group had statistically different rates of grade ≥3 radiation pneumonitis (17.1% *vs*. 8.2%, P=0.034) and grade ≥3 radiation esophagitis (21.6% *vs*. 11.2%, P=0.026), consistent with the aforementioned research results.

Liu et al. (14) found that age, N staging, and T staging were independent prognostic factors for esophageal cancer. This study identified age as an independent prognostic factor, considering that the study population had older age with a higher proportion of comorbidities and non-cancer deaths. Compared to younger patients, most of them were unable to tolerate simultaneous radiotherapy and chemotherapy, which reduced treatment efficacy. Zhang et al. (15) analyzed the survival outcomes of 1349 esophageal cancer patients and considered N staging and T staging as prognostic factors for radiotherapy in esophageal squamous cell carcinoma. Gao et al. (16) demonstrated that gender and synchronous chemotherapy were independent factors affecting patient prognosis (P=0.003, P<0.001). Lyu et al. (17) concluded that clinical staging was an independent predictive factor for overall survival. Similar results were obtained in this study.

For elderly esophageal cancer patients, due to advanced age, poor physical condition, and multiple comorbidities, their tolerance to toxic side effects is reduced. The goal of treatment is to ensure quality of life while prolonging survival as much as possible. The results of this study found no significant differences in survival, efficacy, or treatment failure between the two groups of patients. However, the IFI group had relatively lower toxic side effects. Therefore, for elderly esophageal cancer patients, it may be more appropriate to choose involved field irradiation.

This study is a retrospective study, and it has limitations in terms of lacking real-time follow-up and control, leading to potential information bias and data loss in directly capturing and adjusting for the influence of other factors. In future work, we will improve our research methods by conducting prospective randomized clinical trials and including more potentially related factors for analysis, aiming to provide more reliable clinical diagnostic and therapeutic guidelines for colleagues both domestically and internationally.

## Data availability statement

The original contributions presented in the study are included in the article/supplementary material. Further inquiries can be directed to the corresponding authors.

## Ethics statement

The studies involving humans were approved by the Institutional Review Board of Anhui Medical University. The studies were conducted in accordance with the local legislation and institutional requirements. Written informed consent for participation was not required from the participants or the participants’ legal guardians/next of kin in accordance with the national legislation and institutional requirements.

## Author contributions

YD: Conceptualization, Data curation, Formal analysis, Investigation, Methodology, Validation, Writing – original draft, Writing – review & editing. DH: Data curation, Writing – review & editing. WZ: Data curation, Writing – review & editing. JW: Validation, Writing – review & editing.
